# *Prunus mume* Seed Exhibits Inhibitory Effect on Skin Senescence via SIRT1 and MMP-1 Regulation

**DOI:** 10.1155/2021/5528795

**Published:** 2021-06-01

**Authors:** Hyeong-U Son, Hee-Jeong Choi, Md Badrul Alam, Chae Gyeong Jeong, Hansong I Lee, Solomon L. Kim, Peijun Zhao, Tae-Ho Kim, Sang-Han Lee

**Affiliations:** ^1^School of Food Science and Biotechnology, Graduate School, Kyungpook National University, Daegu 41566, Republic of Korea; ^2^Food and Bio-Industry Research Institute, Inner Beauty/Antiaging Center, Kyungpook National University, Daegu 41566, Republic of Korea; ^3^Department of Chemistry, University of Southern California, Los Angeles, CA 90089, USA; ^4^College of Food Science and Technology, Henan Agricultural University, Zhengzhou, Henan 450002, China; ^5^Biomedical Research Institute, Kyungpook National University Hospital, Daegu 41940, Republic of Korea; ^6^knu BnC, Daegu 41566, Republic of Korea

## Abstract

The *Prunus mume* seed is a by-product of the food industry, and we studied its potential as a food biomaterial, particularly for nutraceutical and inner beauty products. Alternative animal tests showed that an extract of *P. mume* ripened seed (PmRS) was not toxic on the skin. PmRS exhibited protective effects against ultraviolet- (UV-) induced skin aging in mice, confirmed by phenotypic indications, including increased collagen levels and decreased skin thickness. Compared with the UV-saline group, the UV-PmRS group showed increased levels of silent mating type information regulation 2 homolog 1 (SIRT1) and collagen and decreased matrix metalloproteinase- (MMP-) 1 expression. The protective effect of PmRS treatment against UVB-mediated cell viability was observed *in vitro* without any cytotoxicity, and PmRS prevented UVB-induced reactive oxygen species generation in HaCaT cells. PmRS downregulated MMP-1 and MMP-13 compared with the UVB-irradiated group. However, mRNA expressions of tissue inhibitor of metalloproteinase-1 and SIRT1 were upregulated by PmRS treatment. MMP-1 and SIRT1 treated with PmRS were decreased and increased, respectively, at the protein level. Moreover, PmRS treatment reduced c-Jun N-terminal kinase and p38 phosphorylation compared with the UVB-treated group. We postulate that *P. mume* seed could be a useful ingredient in nutraceuticals and inner beauty-purpose foods.

## 1. Introduction

Although aging is a natural phenomenon, people try to reverse it to achieve a healthy and happy life [1]. Skin aging is relevant to social activity because a good appearance improves self-confidence and creates a favorable impression [2]. Although skin senescence occurs with increasing age, it can be affected by ultraviolet (UV) radiation. Furthermore, facial skin senescence differs among individuals of the same age. Skin aging is classified as either intrinsic (chronological) skin aging and/or photoaging. Photoaging is associated with various symptoms, including a thickened epidermis, deep furrows, reddened skin caused by burns, differently pigmented areas, severe skin atrophy, and reduced collagen fibers. Intrinsic skin aging is accompanied by wrinkles, laxity, a dry or rough skin surface, a thin epidermis, and a saggy appearance [3, 4]. Of these two types of skin aging, photoaging is easier to study because of ease of control, rapid stimulation, possible dramatic effects using novel materials, and the low assay cost. Intrinsic skin aging is also affected by photoaging. Thus, the photoaging model was used in this study.

*Prunus mume* Sieb. et Zucc of the family Rosaceae has been cultivated in East Asia, including China, Japan, and Korea, for more than 3,000 years [5, 6]. It is called “Japanese apricot” or “Japanese plum” in Japan, “Chinese mei” in China, and “maehwa” in Korea. Its fruit is called “ume” in Japanese and “maesil” in Korean. The major compound of the polysaccharide fraction of *P. mume* is galacturonic acid [7]. The methanolic extract of the flower buds of *P. mume* can inhibit melanogenesis in B16 melanoma 4A5 cells [8]. Mumeose, an acylated sucrose isolated from the flower buds, has demonstrated an inhibitory effect against aldose reductase [9]. A 70% ethanol extract of *P. mume* fruits repressed glucose uptake in C2C12 cells and regulated glucose intolerance in a high-fat diet-fed mouse model [10], and the leaf extract lowered blood glucose levels in diabetic mice [11]. The ethyl acetate fraction of a *P. mume* 70% ethanol extract inhibited *α*-glucosidase activity and reduced blood glucose levels in obese and diabetic mice [11]. Furthermore, 3,4-dihydroxybenzaldehyde isolated from the *P. mume* seed repressed the damage caused by hydrogen peroxide in granulosa cells [12].

In 2000, the production of *P. mume* fruit was estimated as 7,743 tons from 1,034 hectares of Korean farmlands [13]. According to the statistics from the Ministry of Agriculture, Food, and Rural Affairs in Korea, *P. mume* fruit production in Korea was worth approximately 7.2 million US dollars in 2010, 7.4 million in 2011, 13.9 million in 2012, and 16.7 million in 2013. In 2010, Tsubaki et al. estimated that 500 tons of *P. mume* seed were wasted annually as a by-product in Wakayama Prefecture, a major production area in Japan [14]. Because the dry weight of the seed (including the stone) comprises 40.4% of the whole fruit, it is a plentiful by-product [14]. Therefore, the usefulness of *P. mume* seed as a valuable product warrants investigation. This study focuses on alleviating skin senescence after the application of *P. mume* extracts to the surface of the skin to investigate whether the extracts and amygdalin could inhibit skin aging in a UV irradiation model. We also aimed to collect decisive data for the development of a food ingredient for inner beauty purposes.

## 2. Materials and Methods

### 2.1. Sample Preparation and High-Performance Liquid Chromatography (HPLC)

**Figure 1:**
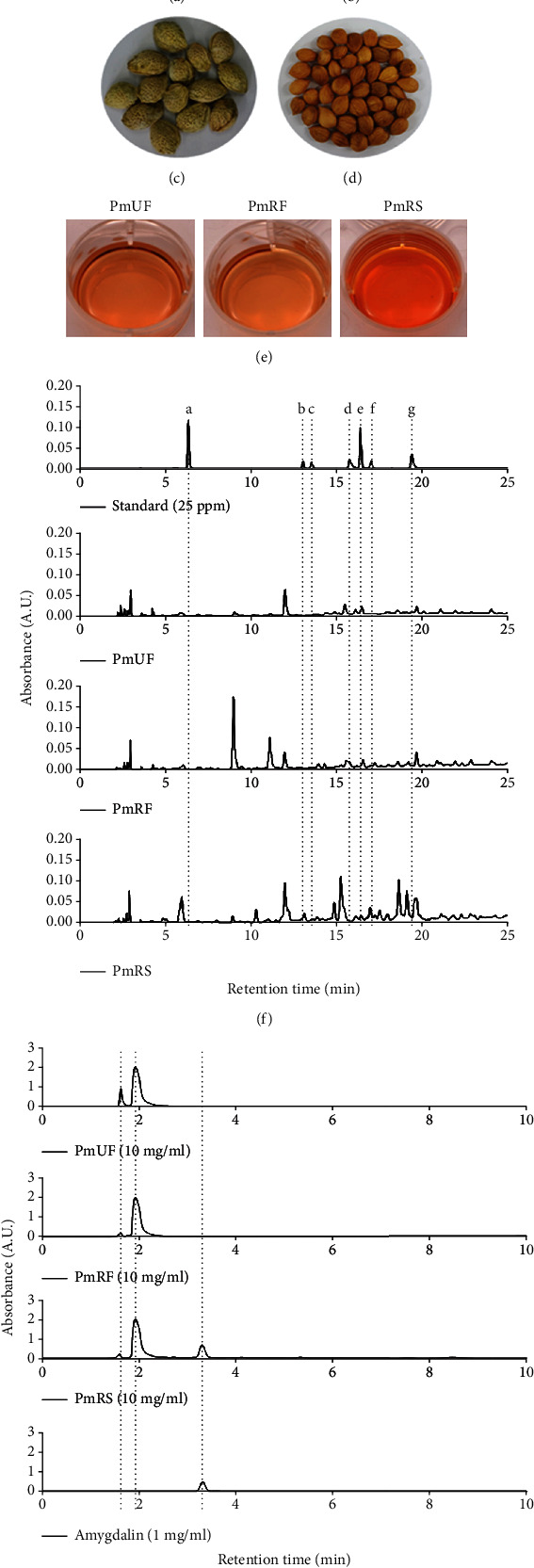
Classical features of the unripe fruit, ripe fruit, and ripe seed of *Prunus mume*. The unripe (a) and ripe (b) fruits were harvested on June 10 and July 12, 2016, respectively, at a local farm (GPS: 35.737666, 129.141598) near Gyeongju, Korea. The ripe fruit was divided into firm outer parts (c) and soft inner parts (d). (e) The aqueous extracts of unripe flesh (PmUF), ripe flesh (PmRF), and ripe seed (PmRS) were dissolved in dimethyl sulfoxide at a concentration of 100 mg/ml. (f) Isocratic analysis was performed to measure the flavonoids. The flow rate was set at 1.5 ml/min with a mobile phase of acetonitrile : distilled water (DW; 20 : 80 *v*/*v*), and absorbance was measured at 218 nm. A: gallic acid, B: (−)-epigallocatechin, C: catechin gallate, D: (−)-epigallocatechin gallate, E: caffeine, F: (−)-epicatechin, and G: (−)-epicatechin gallate. (g) Gradient analysis was performed to measure amygdalin. The flow rate was set at 1 ml/min with a gradient between solvent A (water-acetic acid, 97 : 3 *v*/*v*) and solvent B (100% of methanol). The sample (10 *μ*l) was injected into a C_18_ column maintained at 30°C. For the measurement of flavonoids, the flow rate was set at 1.5 ml/min with mobile phase acetonitrile : DW (20 : 80 *v*/*v*), and absorbance was measured at 218 nm.

*P. mume* fruits were purchased from a farm (GPS: 35.737666, 129.141598) near Gyeongju, Korea, on June 10 (unripe) and July 12 (ripe), 2015 (Figures 1(a)–1(d)). The sample names were abbreviated as *Prunus mume* unripe flesh (PmUF) (50% ethanolic extract of *P. mume* unripe fruit), PmRF (50% ethanolic extract of *P. mume* ripe fruit), and PmRS (50% ethanolic extract of *P. mume* ripe seed). The fruit was divided into flesh and seed and then extracted using 50% ethanol at 65°C in a shaking incubator (Ilshin Biobase, Goyang, Korea) for 24 h. The extracted supernatant was filtered using Whatman No. 1 filter paper (Schleicher & Schuell Bioscience, Keene, NH, USA) and lyophilized (MCTD85; Ilshin Biobase). The voucher specimens of *P. mume* fruit and seed were deposited in the Laboratory of Food Enzyme Biotechnology, Kyungpook National University, Daegu, Korea (2015-pmf and 2015-pms).

The flavonoid content was analyzed on a Waters 2695 HPLC system (Waters, Milford, MA, USA). Briefly, the PmUF, PmRF, and PmRS samples (10 mg/ml) were eluted and filtered through a syringe filter (0.2 *μ*m; Pall Life Sciences, Port Washington, NY, USA). Gallic acid, (−)-epigallocatechin, catechin gallate, (−)-epigallocatechin gallate, caffeine, (−)-epicatechin, and (−)-epicatechin gallate were obtained from Sigma-Aldrich (St. Louis, MO, USA) and used as standards at concentrations of 12.5, 25, 50, and 100 ppm. The sample (10 *μ*l) was injected into a SunFire C18 column (4.6 × 250 mm, 5 *μ*m; Waters). Gradient elution was conducted by varying the proportion of solvent A (water-acetic acid, 97 : 3 *v*/*v*) to solvent B (100% of methanol) at a flow rate of 1 ml/min, and absorbance was measured at 218 nm, as previously reported [15]. For the measurement of amygdalin by HPLC, the three extracts (10 mg/ml) were eluted and filtered through a syringe filter (0.2 *μ*m; Pall Life Sciences). Amygdalin (A6005, Sigma-Aldrich) was used as a standard at concentrations of 10, 50, 100, 200, and 1,000 ng/ml for the quantitative analysis. The samples (10 *μ*l) were injected into a Sep-Pak C18 column (4.6 × 250 mm, 5 *μ*m; Waters) maintained at 30°C The flow rate was set at 1.5 ml/min with a mobile phase of acetonitrile-distilled water (DW; 20 : 80 *v*/*v*), and absorbance was measured at 218 nm.

### 2.2. Antioxidant Assays

The DPPH radical scavenging capacity assay, ferric reducing/antioxidant power assay, and cupric ion reducing antioxidant capacity assay were performed to assess the antioxidant activity of *P. mume*. The concentrations of total phenols and total flavonoids in *P. mume* extracts were determined, as reported previously [16]. Briefly, the total phenol content (TPC) was measured using Folin-Ciocalteu reagent (kept at 25°C) at a ratio of 1 : 10 with the sample [17]. Then, 2 ml of 20% Na_2_CO_3_ solution was reacted for 1 h, and the resultant blue complex was measured at 750 nm. The total flavonoid content (TFC) of each *P. mume* extract was assayed according to a previously described method [18]. Briefly, 2.8 ml of DW was mixed with 0.1 ml of 0.1% (*v*/*v*) potassium acetate solution and 0.1 ml of 10% (*w*/*v*) AlCl_3_, and 0.5 ml of the sample was diluted with 1.5 ml of methanol. Then, the two prepared solutions were mixed together. The mixture was kept at room temperature for 30 min, and the absorbance was measured at 415 nm. The TPC and TFC results were expressed as milligrams of gallic acid or catechin equivalents/100 mg of extract, respectively, as described elsewhere [19].

### 2.3. UV-Irradiated Skin Aging Mouse Model

BALB/c mice (7 weeks old, male, 20–22 g) were purchased from Samtaco Korea, Ltd. (Osan, Korea). The mice were housed in an air-conditioned animal room at 22 ± 1°C with 65% ± 5% humidity. All procedures were performed in compliance with the guidelines of the Committee on Research and Ethical Issues of the International Association for the Study of Pain and the in-house guidelines of Kyungpook National University (KNU-2015-0122). Normal mice were randomly divided into five groups: UV-negative with saline and UV-positive with saline, PmUF, PmRF, and PmRS. To evaluate the inhibitory effect of the *P. mume* samples against photoaging, the mice were shaved with a depilatory cream (Niclean; Il Dong Pharmaceutical Co., Ltd., Seoul, Korea). The sample solution was dissolved in a 1 : 1 volume of 10 ml of saline and 10 ml of 1,3-butylene glycol. After 50 *μ*l of the preparation (25 mg/ml) had been applied for 30 min to the back surface of the mouse, the surface was irradiated with UV rays from a distance of 15 cm using a UV lamp. The UV lamp was set at 1.25 mW/cm^2^ using a UV meter (UV-340; Custom Co., Ltd., Tokyo, Japan). UV radiation was applied to the mice, gradually increasing the doses five times per week for four weeks (1 minimal erythema dose (MED) = 75 mJ/cm^2^: 3 days, 2 MED: 3 days, 3 MED: 4 days, and 4 MED: 10 days).

### 2.4. Measurement of the Physical Properties of Mouse Back Skin Tissue

The thickness of the mouse back dorsal skin tissue was measured using a digimatic thickness gauge. A Sircol Collagen Assay kit (Biocolor Life Science Assays, Carrickfergus, UK) was used to quantify the collagen in the skin. Briefly, skin tissues were homogenized with liquid nitrogen, and then 100 mg of homogenized tissue was dissolved in a pepsin-acid solution (0.1 mg/ml pepsin in 0.5 M acetic acid) at 4°C for 24 h. The solution was centrifuged at 12,000 rpm for 10 min, and the supernatant, which contained the soluble collagen, was isolated. Thereafter, the instructions in the assay kit manual were followed.

### 2.5. Immunohistochemical (IHC) Analysis

Mouse dorsal back skin was isolated after the mice were sacrificed, and IHC analyses were performed with slight modifications according to previously reported methods [20, 21]. Briefly, the tissue was fixed in a 10% formaldehyde solution in PBS for 24 h and then embedded in a paraffin block. Each section was cut into 5 *μ*m thick slices and treated with xylene for deparaffinization. Then, the sections were treated with 3% hydrogen peroxide in a methanol solution to prevent endogenous peroxidase activity, and the epitopes were retrieved. The slides were treated with 10% normal goat serum for 1 h and then incubated overnight at 4°C with antibodies to silent mating type information regulation 2 homolog 1 (SIRT1; ab166821; Abcam, Cambridge, MA), interleukin- (IL-) 1*β* (ab9722; Abcam), and matrix metalloproteinase- (MMP-) 1 (ab137332; Abcam). Finally, the sections were stained with hematoxylin and eosin (H&E) and Masson's trichrome stain, as previously reported [22].

### 2.6. Cell Culture, Cell Viability Assay, and UVB Irradiation

Immortalized human keratinocyte HaCaT cells (AddexBio Technologies, San Diego, CA, USA) were cultured in DMEM supplemented with 10% FBS and 1% penicillin-streptomycin at 37°C in 5% CO_2_. Cell viability was determined using an MTT assay. Briefly, HaCaT cells were cultured in 96-well plates at a density of 1 × 10^5^ cells/well and maintained for 24 h. Once the cells reached a confluency of 80%–90%, they were treated with each concentration of PmRS (10, 30, and 100 *μ*g/ml) for an additional 24 h. Then, the medium was replaced with MTT solution, and formazan crystals were dissolved by adding DMSO. Finally, the absorbance was measured at 595 nm.

For UVB irradiation experiments, HaCaT cells were seeded into 96-well dishes (1 × 10^5^ cells per well) and incubated for 24 h. Various concentrations of PmRS were added to the cells for 24 h, and the media were exchanged with 100 *μ*L of PBS prior to UVB irradiation (40 mJ/cm^2^) using a Bio-Link Crosslinker UV irradiation system (Vilber Lourmat, Marne la Vallée, France). The overall average UVB radiation exposure was 8.01 mJ/cm^2^/d that was equivalent to the daily UVB exposure levels in high-school students measured with digital dosimeters [23]. In this experiment, the total UVB radiation exposure was 40 mJ/cm^2^, which was equivalent to approximately 5 days of sun exposure. The cell culture media were replaced with new media containing predetermined concentrations of PmRS for an additional day. Finally, the MTT assay was performed to measure cell viability.

### 2.7. Measurement of Intracellular Reactive Oxygen Species (ROS)

We used the redox-sensitive dye H_2_DCFDA to determine intracellular ROS levels. Briefly, HaCaT cells were seeded in 96-well black clear-bottomed plates and treated with PmRS for 24 h, followed by UVB exposure (40 mJ/cm^2^) for 24 h. Then, the cells were stained with 25 *μ*M H_2_DCFDA for 1 h at 37°C in the dark. Subsequently, the fluorescence intensity was analyzed at excitation and emission wavelengths of 485 and 535 nm, respectively.

### 2.8. Gene Expression Analysis Using Reverse Transcription-Polymerase Chain Reaction (RT-PCR)

Mouse back dorsal skin tissue was homogenized with liquid nitrogen and treated with TRIzol reagent (Invitrogen, Carlsbad, CA). RT&GO Mastermix (MP Biomedicals, Aurora, OH, USA) and Taq DNA polymerase (Promega Corp., Madison, WI, USA) were used for RT-PCR. The other steps were performed, as previously described [24]. The primer sets used in this study were as follows: SIRT1, forward (F): 5′-GGT AGA GCC TGC ATA GAT CTT CA-3′ and reverse (R): 5′-TGG CAG TAA TGG TCC TAA CTG GG-3′; SIRT2, F: 5′-CCA TGA CCT CCC GCA GGA CAG CG-3′ and R: 5′-GGG TCC CCA GGA AAG GGA GCC TAC-3′; SIRT4, F: 5′-CAC CCG GTC TGA CGA TTT GGC TT-3′ and R: 5′-CCG TGT TAG CTA TTG CTC CTG CC-3′; SIRT6, F: 5′-GGG GAC TGA GCC CAG GTT TGC AT-3′ and R: 5′-CTT CTG GGA GCC TGG GGC CCT TA-3′; SIRT7, F: 5′-TAT CCT AGG AGG CTG GTT TGG CA-3′ and R: 5′-GGA GGC TTA GTT AGA TTC TCC CT-3′; MMP-3, F: 5′-TGG TGG GTG GAT GAG TAA TG-3′ and R: 5′-CGC TGG TAT AAG GTG GTC TG-3′; MMP-8, F: 5′-ATG CAC ATC ACC CTC TGT GA-3′ and R: 5′-CTC TGT GAC CCA GTC CAT CC-3′; MMP-9, F: 5′-GAG GGC CAA GAC GAA GAC ATC-3′ and R: 5′-CAG ATC ACG TCA TCG CAC AAC-3′; MMP-13, F: 5′-CAA GGC TGG TTA CCC AAC AG-3′ and R: 5′-CAC CTG GGA CAA CTG GAA TC-3′; MMP-14, F: 5′-GAG CAC CAA GGT TCT GCT TC-3′ and R: 5′-CTC TCC ATA CGG CGA GAG TC-3′; MMP-17, F: 5′-CAG GAG GAA CTG TCC AAA GC-3′ and R: 5′-GGT TCC TCT TGC TCC ATT TG-3′; and GAPDH, F: 5′-GCG AGA CCC CAC TAA CAT CA-3′ and R: 5′-GAG TTG GGA TAG GGC CTC TCT T-3′. After the reaction process (18–34 cycles of 30 s at 95°C, 30 s at 56°C, and 45 s at 72°C), each PCR product was analyzed using 1% agarose gel electrophoresis with ethidium bromide staining. The PCR product was confirmed by comparison with a 1 kb DNA ladder (Solgent, Daejeon, Korea). The relative intensity of the PCR products in each set was standardized using a ChemiDoc XRSþ image analyzer (Bio-Rad, Hercules, CA, USA) for semiquantitative analysis.

### 2.9. Western Blot Analysis

Western blot analysis was performed with slight modifications, as previously described [25]. The primary antibodies for SIRT1, IL-1*β*, and MMP-1 were the same as those used in the IHC investigations. The band intensities were calculated using a ChemiDoc XRS+image analyzer (Bio-Rad). The antibodies of filaggrin (SC-80609, Santa Cruz Biotechnology, Santa Cruz, CA, USA) and COX-2 (BS1076, Bioworld Technology, Bloomington, MN, USA) were commercially available.

### 2.10. Statistical Analysis

Data were expressed as the mean ± standard deviations. Statistical significance was determined by one-way ANOVA with Tukey *post hoc* tests using SPSS v21.0 software (IBM Corp, Chicago, IL, USA). *P* values < 0.05 were considered statistically significant [26].

## 3. Results

### 3.1. Characteristics and Antioxidant Activities of the P. mume Extracts

**Figure 2:**
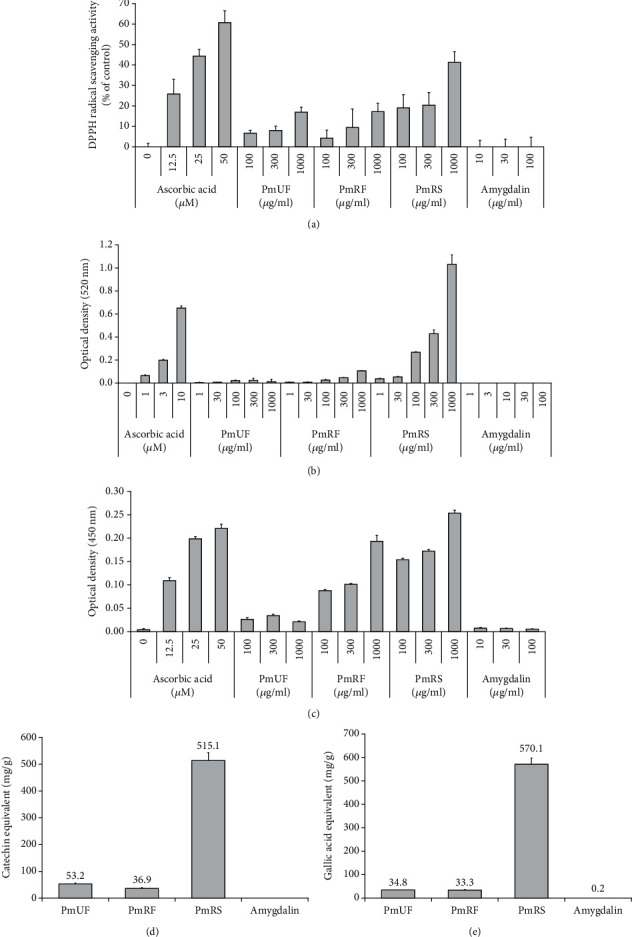
Analysis of the antioxidant capacities of the aqueous extracts of *P. mume* unripe flesh (PmUF), ripe flesh (PmRF), and ripe seed (PmRS). (a–c) Measurement of antioxidant potentials. DPPH assay (a), ferric reducing/antioxidant power assay (b), and cupric ion reducing antioxidant capacity assay (c) were performed to measure the antioxidant capacities of the extracts. (d, e) Determination of total phenol content and total flavonoid content. Total phenol content (d) and total flavonoid content (e) were also measured, as described in detail in the Materials and Methods section.

HPLC analysis was performed using several standard antioxidant materials to determine the active ingredients of the *P. mume* extracts. We categorized the samples as unripe flesh (PmUF), ripe flesh (PmRF), and ripe seed (PmRS), all of which displayed classical features (Figures 1(a)–1(c)). The ripe fruit was divided into firm outer parts (Figure 1(c)) and soft inner parts (Figure 1(d)). The inner parts were used for further study (Figure 1(e)). HPLC showed three unique peaks in PmRS (Figure 1(g)) with the retention times as follows: peak 1 (1.60 min), peak 2 (1.95 min), and amygdalin (3.27 min; 149.29 *μ*g/mg). Following HPLC, we determined the flavonoids in the extracts by comparing the data, which showed several peaks (Figure 1(f)). A comparison between the unripe and ripe fruit revealed that two peaks, at a retention time of 8.97 and 11.09 min, respectively, were considerably increased after ripening. Additionally, PmRS showed many more specific peaks compared with other the samples, and these were expected to be antioxidant compounds. Analysis of the antioxidant capacities of the aqueous extract of PmUF, PmRF, and PmRS revealed that PmRS had the most potent radical scavenging abilities compared with than that of PmUF or PmRF (Figure 2). These results indicated that the three designated extracts could potentially mediate antioxidant activities. Therefore, we further investigated the antiaging effect on skin by confirming the molecular mechanisms involved.

### 3.2. Aged and Damaged Skin Appearance from UV Irradiation and the Preventive Effects of P. mume Seed Extract

**Figure 3:**
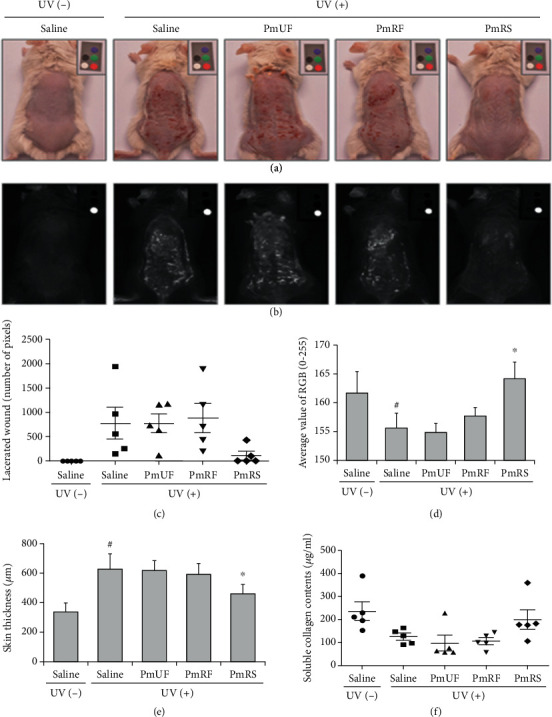
Phenotypic comparison of the mice skin surfaces with or without ultraviolet (UV) irradiation. The back skin of the BALB/c mice was photographed after UV irradiation, and 50 *μ*l of the sample (25 mg/ml) was applied 20 times. Raw photographs were taken using a digital camera with a color marker (a). The photographs from (a) were processed to invert the red color so that the red color on the manufactured color marker and the damaged skin area were converted and shown as a bright color (b). To calculate damage to the skin, the ripped skin was transformed from a binary color image into a black and white image (c). Redness was calculated using ImageJ software according to a histogram of RGB values (d). Back skin thickness was directly calculated using a digimatic thickness gage (e). Collagen content was determined from skin lysates using a Sircol Collagen Assay Kit (f). Results are shown as the mean ± SD. ^#^*P* < 0.05 compared with the UV-nonirradiated group. ^∗^*P* < 0.05 compared with the UV-irradiated group. PmUF: *Prunus mume* unripe fruit; PmRF: *P. mume* ripe fruit; PmRS: *P. mume* ripe seed.

UV irradiation caused several types of phenotypic damage to the skin. First, the mice exhibited local erythema after 20 UV irradiations over 4 weeks (Figure 3(a)). To compare erythematic damage or skin redness on the mouse dorsal skin treated with the *P. mume* extract, the photographs were processed to invert the red color value in the picture (Figure 3(b)). Among the sample treatment groups, erythema was clearly alleviated in the PmRS group. Damage and senescent skin symptoms with deep, red-colored lacerated wounds and surface redness were also observed (Figure 3(a)). The symptoms were more clearly observed after image processing using red color standardization (Figure 3(b)). However, there is no correlation between redness of skin and back skin thickness which was related to skin edema.

### 3.3. Determination of Skin Morphologies in the UV-Irradiated Mouse Model

Because an analogous scar and a striking red color were observed, we next tested whether prolonged UV irradiation caused phenotypic skin changes other than wrinkles, which were marked as lacerated wounds (Figure 3(c)). Furthermore, the color tone of the whole skin was calculated as low brightness by skin burn using ImageJ software (Figure 3(d)). These results showed that the extracts had no cytotoxicity from UV irradiation under the designated conditions.

**Figure 4:**
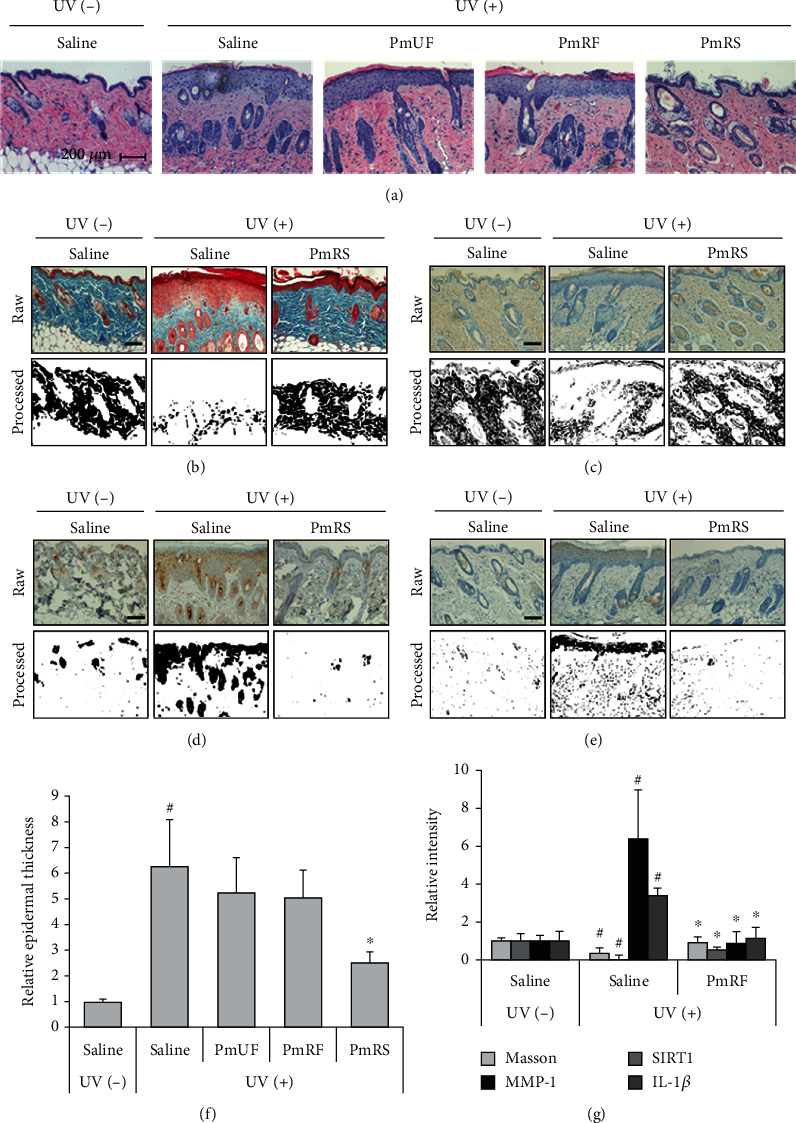
Immunohistochemical analysis of ultraviolet- (UV-) irradiated skin tissue. Mouse back skin tissue was fixed with 10% formaldehyde and embedded in paraffin. Stained slides were observed using an Eclipse TE-2000U inverted microscope and camera (Nikon, Tokyo, Japan). (a) Hematoxylin and eosin (H&E) staining. (b) Masson's trichrome staining to determine the collagen content. (c–e) Expressions of skin senescence-related biomarkers. SIRT1 (c), MMP-1 (d), and IL-1*β* (e) were detected by antibodies using immunohistochemistry. (f) Measurement of epithermal thickness. Epidermal thickness was calculated from the H&E results using ImageJ software. (g) Comparison of relative intensity of the collagen, SIRT1, MMP-1, and IL-1*β*. The intensity was calculated by the above (b–e) images. Results are shown as the means ± SD. ^#^*P* < 0.05 compared with the UV-nonirradiated group. ^∗^*P* < 0.05 compared with the UV-irradiated group. PmUF: *Prunus mume* unripe fruit; PmRF: *P. mume* ripe fruit; PmRS: *P. mume* ripe seed.

The skin color tone of the PmRS-treated group was similar to that of the UV-untreated group (Figure 4(d)). A designated area from the photographs provided various types of information, and the RGB value was displayed as a histogram according to the color pixel distribution. The normal skin color tone of the C57BL/6 mice was 161.7 ± 3.7, but UV irradiation decreased the value to 155.6 ± 2.6, indicating a change to a darker tone. The skin tone of the PmUF group was 154.9 ± 1.5, that of the PmRF group was 157.7 ± 1.5 (similar to the UV(+)/saline group), and that of the PmRS group was 164.1 ± 2.8 (similar to the UV-negative group) (Figure 3(d)).

### 3.4. Determination of Skin Elasticity and Collagen Content

We used a mouse model to test whether the extracts exhibited an antiaging effect on the skin. The mean thickness of normal mouse skin was 0.337 ± 0.057 mm, whereas the mean thickness of UV-irradiated skin was 0.624 ± 0.106 mm, representing an increase of approximately 84.91 ± 31.37% (Figure 3(e)). The mean skin thickness in the PmRS group was 0.460 ± 0.060 mm, which was a 26.17 ± 9.61% decrease compared with the UV-positive control. Overall, we collected significant data on the protective effect of PmRS on skin aging in the mouse model.

### 3.5. IHC Analysis Showed an Alleviation Effect in the PmRS Group

IHC analysis was performed to determine the regulation of senescence by UV irradiation using formalin-fixed paraffin-embedded tissue. Similar to the results presented in Figure 3(e), the UV-irradiated group had an increased epidermal thickness compared with the negative control group (Figure 4(a)). Five photographs were randomly selected, and the average epidermal thickness was calculated as the length proportion of the epidermal area using ImageJ software. The epidermal thickness in the UV-irradiated group was approximately six times greater than that in the nonirradiated group, whereas the epidermal thickness in the PmRF group was decreased by 60% compared with that of the UV-positive control (Figure 4(f)). Masson's trichrome staining on the IHC slide showed blue-colored collagen in the dermis (Figure 4(b)). The UV-irradiated group displayed decreased collagen content throughout the outer dermis, whereas the collagen content in the PmRS group was similar to that in the normal group. The collagen-positive area was calculated in the processed photographs as the pixel number using ImageJ software. The collagen-positive area in the UV-irradiated group was decreased compared with those in the UV-negative group and the PmRS group (UV(−): 100%, UV(+): 35%, and PmRS: 94%; Figure 4(g)). SIRT1 was distributed throughout the dermis, showing slightly brown staining. Unfortunately, the SIRT1 staining pattern was difficult to observe in the IHC analysis, so its photographs were transformed into a binary pattern in black and white color (Figure 4(c)). The image threshold was determined using ImageJ software, and all photographs were processed under the same conditions. The results indicated that SIRT1 activation in the UV-irradiated group was dramatically decreased, but this was alleviated in the PmRS group (UV(−): 100%, UV(+): 12%, and PmRS: 56%; Figure 4(g)). In sequence, MMP-1 (Figure 4(d)) and IL-1*β* (Figure 4(e)) were also stained with brown color, and staining was primarily distributed in the epidermis. Additionally, MMP-1 (UV(−): 100%, UV(+): 535%, and PmRS: 86%; Figure 4(g)) and IL-1*β* (UV(−): 100%, UV(+): 340%, and PmRS: 120%) were positively regulated in the PmRS group (Figure 4(g)).

### 3.6. SIRT and MMP Regulation by PmRS in UV-Irradiated Mice

**Figure 5:**
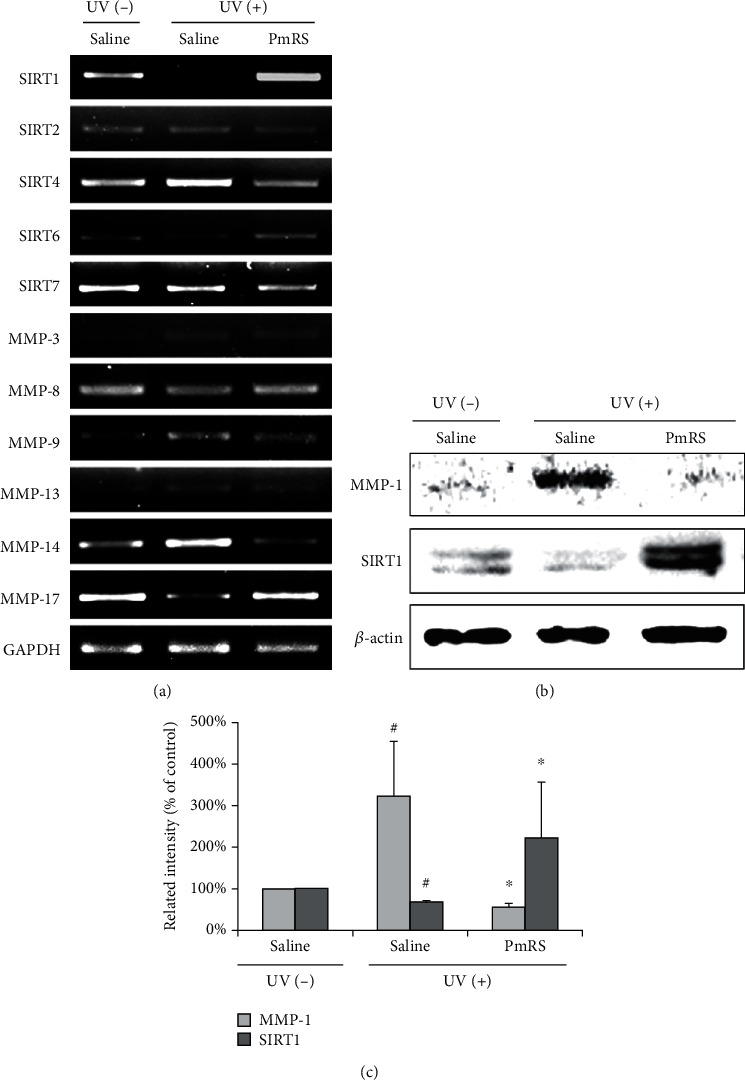
Detection of gene expression of SIRTs and MMPs in mouse skin tissues using reverse transcription-polymerase chain reaction (RT-PCR) and western blots. Mouse back skin tissues were isolated after undergoing ultraviolet (UV) irradiation 20 times. (a) RNA was isolated from skin tissue using TRIzol reagent, and RT-PCR was performed to detect RNA gene expression of SIRTs and MMPs. (b, c) Tissue lysate (0.1 mg) was loaded for western blots. Band intensities were calculated using the ChemiDoc XRS+ Image Lab software (Bio-Rad). Results are shown as the means ± SD. ^#^*P* < 0.05 compared with the UV-nonirradiated group. ^∗^*P* < 0.05 compared with the UV-irradiated group. PmRS: *Prunus mume* ripe seed. Results of RT-PCR and western blots are shown as a classical set of 3 independent experiments.

The expressions of SIRTs and MMPs were investigated to determine how the sample extracts alleviated skin aging after exposure to UV irradiation. SIRT1 RNA gene expression in the UV-irradiated group was decreased by 76% compared with that in the normal group, whereas SIRT1 RNA gene expression was increased in the PmRS group (Figure 5(a)). Additionally, the SIRT1 protein level was positively regulated in the PmRS group (Figures 5(b) and 5(c)). However, other SIRTs (SIRT2, 4, 6, and 7) and MMPs (MMP-3, 8, 9, 13, 14, and 17) were not significantly regulated, and especially MMP-17 was decreased after UV exposure (Figure 5(a), rows 2–11). These results indicated that MMP-1 and SIRT1 were downregulated on MMP-1 expression as well as upregulated on SIRT1 expression in UVB-induced PmRS-treated tissues.

### 3.7. Effect of PmRS on UVB-Induced Skin Aging-Related Biomarkers in HaCaT Cells

**Figure 6:**
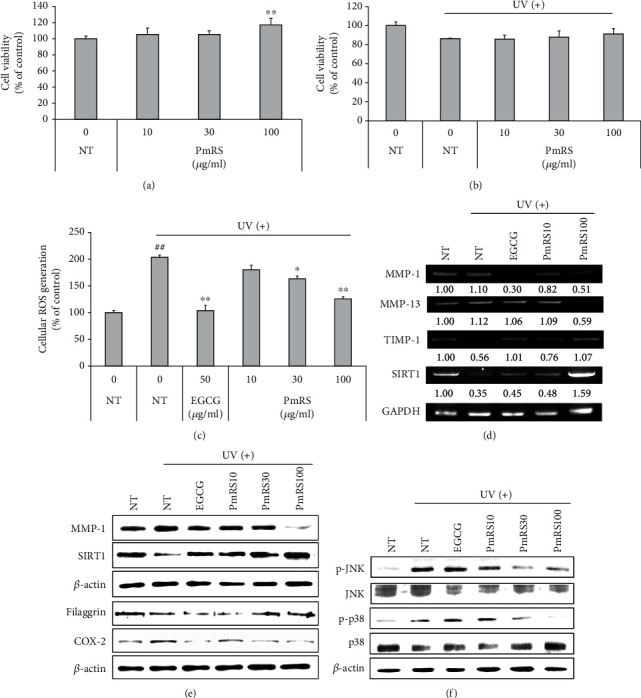
Effect of *P. mume* ripened seed (PmRS) treatment on mitogen-activated protein kinase (MAPK) signaling in HaCaT cells. The effect of PmRS (10, 30, and 100 *μ*g/ml) on cell viability in HaCaT cells (a). Protective effect of PmRS on cell viability (b) and intercellular reactive oxygen species (ROS) generation in ultraviolet- (UV-) B-induced (40 mJ/cm^2^) HaCaT cells (c). Reverse transcription-polymerase chain reaction (d) and western blot analysis (e) of PmRS on skin photoaging-related markers. Phosphorylation of the MAPK signaling pathway in UVB-induced HaCaT cells (f). NT: no treatment; EGCG: epigallocatechin gallate; PmRS10: PmRS, 10 *μ*g/ml; PmRS30: PmRS, 30 *μ*g/ml; PmRS100: PmRS, 100 *μ*g/ml. Results of RT-PCR and western blots are shown as a classical set of 3 independent experiments.

The MTT assay was performed to determine whether PmRS was toxic to HaCaT cells. A concomitant change in the amount of formed formazan indicating the degree of cytotoxicity caused by UVB rays estimated an increase or decrease in viable cells. No cytotoxicity was observed at concentrations of 10, 30, and 100 *μ*g/ml (Figure 6(a)), and these nontoxic concentrations were used to assess the protective effect of PmRS in HaCaT cells. We found that PmRS protected cells from UV-induced death in a dose-dependent manner (Figure 6(b)). An H2DCFDA ROS detection assay was performed to further evaluate whether PmRS could inhibit the generation of UVB-induced ROS. The results revealed that the increased production of intracellular ROS stimulated by UVB irradiation was significantly decreased by PmRS treatment in a dose-dependent manner (Figure 6(c)). The inhibitory effects of PmRS treatment on UVB-induced MMP mRNA levels in HaCaT cells were also detected, thereby proving the antiphotoaging effects of PmRS. Furthermore, RT-PCR revealed that the UVB-induced expression of MMP-1 and MMP-13 was suppressed in the PmRS-treated group (Figure 6(d)). However, UVB-induced reductions of tissue inhibitor of metalloproteinase- (TIMP-) 1 and SIRT1 were upregulated by PmRS treatment (Figures 6(d) and 6(e)). Filaggrin and COX-2 expressions were displayed perfectly by increasing or decreasing their expressions according to the activity (Figure 6(e), 4^th^ and 5^th^ columns). Additionally, western blot analysis definitively showed that UVB exposure increased MMP-1 expression, whereas PmRS treatment reversed this outcome. In contrast, high expression levels of SIRT1 in the PmRS-treated group dramatically increased the SIRT1 protein levels. We assessed the effects of PmRS on mitogen-activated protein kinase (MAPK) phosphorylation (p38, c-Jun N-terminal kinase (JNK), and extracellular signal-regulated kinase (ERK)) in HaCaT cells to further investigate whether PmRS could inhibit UVB-induced MAPK phosphorylation. Our results showed that PmRS inhibited JNK and p38 phosphorylation in a dose-dependent manner (Figure 6(f)), whereas ERK phosphorylation was not significantly affected by PmRS. Together, these data strongly indicated that PmRS exhibited potent potential in mediating SIRT1 upregulation as well as MMP-1 downregulation via inhibition of p38 and JNK phosphorylation in HaCaT cells.

## 4. Discussion

We used a UV irradiation model to investigate whether the extracts of *P. mume* fruit and amygdalin would inhibit skin aging in an effort to develop an ingredient for functional food or inner beauty purposes. It was estimated in 2010 that 500 tons of *P. mume* seed are wasted annually as a by-product in Japan [14]. Furthermore, because the weight of the seed (including the stone) is up to 40% of the whole fruit, it is a plentiful by-product [14]. Therefore, the surplus of *P. mume* fruit and its seed are considered a starting point for the development of processed foods in Korea [13].

We used the peak areas of standard concentrations in an attempt to identify two unknown compounds in PmUF, PmRF, and PmRS chromatograms. Novel flavonoids have been identified from the methanol extract of *P. mume* fruits: 2*β*,3*β*-epoxy-5,7,4′-trihydroxyflavan-(4*α*→8)-epicatechin, 2*β*,3*β*-epoxy-5,7,3′,4′-tetrahydroxyflavan-(4a→8)-epicatechin, isoquercitrin, rutin, quercetin 3-O-neohesperidoside, and (−)-epicatechin [27]. From the results of our HPLC analysis (Figure 1(f)), the unidentified peaks are cautiously expected to be novel flavonoids, such as the abovementioned compounds. The potential candidates include intermediate dietary fibers, such as secondary metabolites of soluble dietary fiber, lignin, cellulose, hemicellulose, and total/acid-soluble pectin, although further analysis is necessary to elucidate the compounds.

Humans have attempted to conquer the two types of skin aging, namely, intrinsic (chronological) aging and extrinsic aging, to lead a healthy life. Of these two, extrinsic aging is controllable and is affected by smoking, environmental pollutants, and UV exposure [28, 29]. Current global interest is moving toward the use of botanical products, and the buzzword in the food and cosmetic industries is “natural” [30]. We investigated antiaging skin ingredients that are natural substances and found several potent materials, including *P. mume*.

To calculate damage to the skin, ripped skin was transformed from a binary color image to a black and white image. Thereafter, redness was calculated using ImageJ software according to a histogram of RGB values. Even though there was a high standard deviation among the lacerated wound values, UV-negative and UV-positive skins were completely different (Figure 3(c)). To compare the lacerated wounds in each photograph, the same pixel size was selected from the raw photographs (25,668 pixels). Thereafter, the wound-positive area was isolated, and the number of pixels was calculated. Although the standard deviations of the pixel numbers of the lacerated wound areas were large, a regular pattern was observed. The UV-negative group rarely had lacerated wound areas (0.8 ± 1.8 pixels), whereas the UV-positive group had large lacerated wound areas (771.6 ± 723.1 pixels). Although a statistically significant difference was not observed compared with the UV-positive group, the PmRS extract exhibited a highly protective effect against wound production on a mouse skin (PmUF: 768.8 ± 433.9, PmRF: 881.0 ± 670.3, and PmRS: 110.6 ± 185.7 pixels of lacerated wound area; Figure 3(c)).

On the other hand, the skin of the UV-irradiated group was less elastic and had a rough texture and more wrinkles compared with the skin of the nonirradiated group, even before the mice were sacrificed at the end of the experiment. These symptoms indicated that collagen was degraded and had collapsed. Interestingly, a mouse dorsal skin became thicker after UV irradiation. Wrinkles are the most important indicator of skin aging and are caused by collagen degradation. Therefore, the amount of collagen in skin tissue was calculated using a Sircol soluble collagen assay kit. Figure 3(f) indicates only soluble collagen, which was extracted with a pepsin-acetic acid solution using the Sircol collagen assay kit. The soluble collagen content was calculated as *μ*g extracted collagen/ml solution. The amount of soluble collagen in the UV-negative group was 237.0 ± 91.1 *μ*g/ml, whereas that in the UV-positive group was 127.0 ± 31 *μ*g/ml, indicating a reduction of approximately 54%. The PmUF group (99.2 ± 73.7 *μ*g/ml) and the PmRF group (107.0 ± 33.6 *μ*g/ml) exhibited values similar to the UV-positive group, whereas the values for the PmRS group (201.4 ± 94.9 *μ*g/ml) were similar to those of the UV-negative group (Figure 3(f)). In this study, the amount of collagen in the skin was not significantly different among the groups, even after the skin was UV-irradiated 20 times. This could be because the Sircol kit only evaluated soluble collagen that was extracted by the pepsin-acetic acid solution. Additionally, the kit may have detected cleaved collagen, which was already degraded by a collagenase such as MMP-1. Therefore, another protocol is required to determine the amount of collagen in the skin.

Skin thickness and epidermal and dermal thickness are commonly examined and considered as symptoms of skin aging [31, 32]. The variance in skin thickness after UV irradiation is influenced by UV power conditions, called the MED. Even so, this symptom is certainly related to skin aging or inflammation. Skin thickness is related to age in both mice and humans. An evaluation of 170 female individuals showed that skin thickness of the forehead, cheeks, and corners of the eyes, which are exposed to sunlight on the face, decreased with age. Skin elasticity was also decreased with age in these skin regions [33]. Gniadecka and Jemec studied the relationship between chronological aging/photoaging and skin thickness in 90 individuals aged 18–94 years. Their results indicated a tendency toward a thicker skin in the subepidermal region of the skin of the dorsal forearm and ventral forearm with age [34].

Skin aging is caused by collagen degradation via MMP-1 activation, and our results show significant variations in soluble collagen content and MMP-1 expression in the skin tissues after UV stimulation. The factors regulating aging at the general cellular level, not restricted to the skin, were evaluated. SIRT1 has been studied for its effect on the aging mechanism. In particular, Gomes et al. determined that SIRT1 is affected by nuclear nicotinamide adenine dinucleotide (NAD^+^) content in the oxidative phosphorylation system and that restoring mitochondrial dysfunction is possible [35]. Therefore, it was suggested that the effects of aging could be prevented and reversed by active natural compounds. SIRT1 is an NAD^+^-dependent class III histone deacetylase that regulates apoptosis, cell senescence, and cell growth by controlling p53, forkhead transcription factors, and PPAR-*γ* [36]. Resveratrol (3,5,4′-trihydroxystilbene), a SIRT1 activator that is abundant in the skin of grapes, has been shown to extend the lifespan of aged mice and positively regulated aging-related factors by reducing insulin-like growth factor-1 and increasing AMP-activated protein kinase and peroxisome proliferator-activated receptor-*γ* coactivator 1*α* [37].

In the field of related skin aging research, SIRT1 has rarely been considered because skin aging is primarily controlled by MMP-1 and collagen regulation [38]. However, recent studies have evaluated SIRT gene regulation in skin aging. Predictably, SIRT1 was positively associated with skin senescence, and SIRT1 and SIRT6 were downregulated in passaged human dermal fibroblasts [39]. Additionally, SIRT6-deficient mice displayed downregulated alpha-1 type I collagen (COL1A1) gene expression and upregulated MMP-1 expression through the activation of nuclear factor kappa-light-chain-enhancer of activated B cells [40]. According to previous reports, only SIRT1, SIRT6, and rarely SIRT7 in the sirtuin family have been associated with dermatologic issues, such as skin aging, skin inflammation, autoimmune disease, cutaneous infection/cancer, and inherited dermatologic disease [41]. Therefore, we evaluated whether severe UV irradiation influenced the gene expression of SIRTs, including SIRT2, SIRT3, SIRT4, and SIRT5. SIRT expression was significantly altered after UV irradiation to the skin (Figure 6(a)). In particular, SIRT1 expression in HaCaT cells revealed that PmRS treatment markedly increased the reduced SIRT1 expression caused by UVB irradiation at the mRNA and protein level (Figure 6). However, the expression of SIRT3 and SIRT5 mRNA was not detected in the skin.

As mentioned above, collagenase, particularly MMP-1 (collagenase-1), is the MMP that is most strongly influenced by UV stimulation and is directly related to skin aging. Similarly, MMP-8 (collagenase-2) and MMP-13 (collagenase-3) are members of the collagenase family and degrade type I, II, and III collagens [42]. Although MMP-2 and MMP-9 (gelatinase B) are factors studied in inflammation and cancer, they are not vastly important in skin aging reactions [43]. One report trivially mentioned that acute UV irradiation increased MMP-1, MMP-3, and MMP-9 in the human skin [44]. Messenger RNA expression of MMP-12 (macrophage elastase) was increased by UVA induction, and MMP-10 (stromelysin-2) was increased by UVB induction in human skin [45]. Additionally, increased expression of MMP-7 at the early stage was detected by IHC analysis in photo-induced rat skin [46]. Interestingly, Quan et al. reported that mRNA levels of MMP-1, MMP-3, and MMP-9 were upregulated 24 h after UV irradiation. In particular, MMP-9 was expressed earlier (at 8 h) in the human epidermis. Furthermore, the expression of MMP-1, MMP-3, and MMP-9 was three to four times higher in the epidermis than in the dermis [47]. In that study, mRNA expression of MMPs was not significantly different after UV irradiation, but MMP-17 was reduced in the UV-positive group (Figure 5(a)). These data collectively demonstrated that PmRS induced a UV-inhibitory event and that our data can be used to support a food or cosmetic ingredient or application, such as a whole food extract against skin inflammation and/or aging. RT-PCR and western blot analysis demonstrated that PmRS significantly reduced the MMP-1 expression in HaCaT cells that had been irradiated with UVB (40 mJ/cm^2^). Furthermore, the mRNA level of MMP-13 was decreased at high concentrations of PmRS (100 *μ*g/ml) in the presence of UVB irradiation. These findings suggest that PmRS has a potential antiphotoaging effect by downregulating MMP production in UVB-irradiated HaCaT cells.

Collagen breakdown leads to wrinkle formation and skin dryness. However, TIMPs downregulate collagen breakdown by irreversibly binding to MMPs, resulting in inactivation. Thus, TIMPs maintain collagen homeostasis by retaining the collagen content of the skin. Therefore, an imbalance between MMP and TIMP expression causes skin damage and pathological symptoms [48]. Thus, MMP inhibition due to TIMP overexpression is considered a major target for treating UVB radiation-mediated skin aging [49]. In our study, a significantly higher level of TIMP-1 mRNA expression was shown in the PmRS100-treated group.

UVB-induced production of ROS in the skin could stimulate MAPK signal transduction [50]. The pathway of MAPK signal transduction plays a critical role in regulating immune response and inflammation, including the expression of MMPs [51]. UVB inhibits the MAPK signaling pathways and MAPK phosphorylation. MAPKs consist of the serine and tyrosine kinase families, namely, ERK, JNK, and p38. UVB radiation-induced ROS generation activates the MAPKs, which are specific to serine and threonine and play a vital role in UVB-induced skin photodamage [52]. Collagen degradation is generally regulated by MMPs through the activity of TIMPs, their natural inhibitor. The phosphorylation of JNK and p38 expression levels stimulated by UVB were clearly downregulated by PmRS treatment.

## 5. Conclusions

The seed extract of *P. mume* was prepared using unripe fruit, ripe fruit, and seed fractions by extraction with 50% ethanol. First of all, extract of *P. mume* ripe seed (PmRS) had the most potent antioxidant effects compared with that of unripe or ripe fruit of *P. mume*. There is no cytotoxicity up to 100 *μ*g/mL of PmRS, and PmRS can regulate the UV-induced cell viability. Furthermore, PmRS reduced ultraviolet (UV) irradiation-induced ROS elevation. It was also able to suppress the UVB-induced MMP-1 and 13 elevations at both activity and mRNA levels. The gene expressions of TIMP-1 and SIRT1 were increased in the PmRS-treated group. Western blot analysis revealed that the PmRS attenuated the MMP-1, while it enhanced the expression of SIRT1. For in vivo assay, the samples were applied to a mouse back skin before UV exposure. The results indicated that the extract of *P. mume* ripe seed (PmRS) exhibited potent protective effects, as indicated by an increase of up to 38% in skin collagen levels and a 27% decrease in skin thickness compared with the UV-irradiated group. The groups to which unripe and ripe fruit extracts were applied exhibited results similar to those of the UV-irradiated group. The skin of the PmRS-treated group was visually close to that of a normal skin, as indicated by lacerated wounds and the color tone of the skin. An IHC assay was performed, and the H&E stain results indicated that in comparison with the UV-irradiated group, the PmRS group exhibited a 60% decrease in skin epidermal thickness and collagen degradation was abated, as indicated by Masson's trichrome staining. PmRS positively regulated SIRT1 (upregulation) and MMP-1 (downregulation), as indicated by RT-PCR, western blots, and IHC.

It appears that *P. mume* seeds can be useful ingredients for skin cosmetic products. The alternative animal tests confirmed that the components in *P. mume* seeds can be used in the cosmetic industry as well as for inner beauty or cosmeceutical purposes.

## Data Availability

The data used to urge the findings of this study are available from the corresponding author upon request.
